# A Comparative Study of Two Bone Graft Substitutes—InterOss^®^ Collagen and OCS-B Collagen^®^

**DOI:** 10.3390/jfb13010028

**Published:** 2022-03-09

**Authors:** Gaurav Jain, Dylan Blaauw, Steve Chang

**Affiliations:** Department of Research and Development, SigmaGraft Inc., 575 Sally Place, Fullerton, CA 92831, USA; dylanoblaauw@gmail.com (D.B.); stevechang@sigmagraft.com (S.C.)

**Keywords:** bone, hydroxyapatite, collagen, bone graft substitute, bone fillers

## Abstract

Bone is a complex hierarchical tissue composed of organic and inorganic materials that provide structure, support, and protection to organs. However, there are some critical size defects that are unable to regenerate on their own and therefore require clinical repair. Bone graft substitutes allow repair by providing a temporary resorbable device. Among the common filler materials that aid in regeneration is hydroxyapatite particles of either animal or human origin which is used to fill or reconstruct periodontal and bony defects in the mouth. However, particulate graft substitutes suffer from localized migration away from the implantation site, necessitating the use of a barrier membrane. In this study, we designed InterOss Collagen, combining bovine hydroxyapatite granules with porcine-skin derived collagen to form a bone filler composite. Physiochemical properties of InterOss Collagen and a commercially available product, OsteoConductive Substitute-Bovine (OCS-B) Collagen, referred to as OCS-B Collagen, were examined. We found two bone graft substitutes to be mostly similar, though InterOss Collagen showed comparatively higher surface area and porosity. We conducted an in vivo study in rabbits to evaluate local tissue responses, percent material resorption and bone formation and showed that the two materials exhibited similar degradation profiles, inflammatory and healing responses following implantation. Based on these results, InterOss Collagen is a promising dental bone grafting material for periodontal and maxillofacial surgeries.

## 1. Introduction

Bone loss in the weeks following tooth extraction is a common problem faced in the field of implant and restorative dentistry [1]. In addition, the trauma and infection associated with this invasive procedure causes alteration to the underlying bone structure. Studies indicate up to 1–3 mm in alveolar ridge height and up to 3–5 mm in width may be resorbed during the healing process [2,3,4]. This loss in bone has severe consequences in terms of potential implant support and overall oral health.

One of greatest advances in dental bone regenerative dentistry is the ability to replace damaged or missing teeth through implants and prosthetic crowns [5,6,7]. However, before the implants can be placed in the defect site, sufficient bone volume is often needed to provide long term stabilization of the implant. 

Bone grafting is a well-established method used to fill or repair the bone defect and replace the lost bone [8,9]. Implantation of resorbable bone substitutes reduces the size of the defect that needs to be mended and provide mechanical support until the tissue has naturally regenerated and remodeled [10,11,12,13]. Among various type of bone substitutes available, particulate bovine bone grafts are one of the most common types of bone substitutes used in dental applications [14]. These materials aim towards filling, augmenting or reconstructing periodontal or bony defects as they offer advantages of dense packing into the irregular and non-uniform defect sites [15]. Calcium phosphates, synthetic and animal origin are the most common types of bone material substitutes commercially available [16,17]. These include Hydroxyapatite, α-tricalcium phosphate (α-TCP), and β-tricalcium phosphate (β-TCP) or a combination mixture forming a composite [18,19,20,21]. These materials exhibit intrinsic porosity and can be tailored to form scaffolds to allow space for the recruitment and proliferation of osteogenic cells. In addition, the degradation products and released ions enhance cell activity and accelerate bone repair. 

However, particulate bone fillers suffer from localized migration from the defect site following implantation attributable to external compressive forces (biting and chewing) in the oral cavity. To overcome this problem, a barrier membrane is often used to cover the defect and contain the particulate grafts preventing its collapse. This increases the cost as well as risk to infection while causing inconvenience and discomfort to the patient.

The two main components of adult human bone are hydroxyapatite and collagen. Collagen, though biocompatible and biodegradable, exhibits sub-optimal mechanical properties and is usually cross-linked or mixed with other proteins, polymers and inorganic materials to develop into an effective bone graft. A composite scaffold combining the advantages of particulate grafts with biopolymers, such as proteins, is often used to overcome the limitations of the use of barrier membrane and contain particulate migration [22]. Several composite bone grafts are commercially available that combine Collagen Type I, the most abundant protein in human body, with bovine hydroxyapatite granules in various ratios to form into spongious scaffolds with an interconnected pore structure allowing easier migration of bone regenerative cells [23,24,25,26]. Bone predominantly constitutes of calcium phosphate (non-stoichiometric carbonate- substituted hydroxyapatite crystals) embedded in collagen matrix. The two components are arranged in a hierarchical structure that defines the chemical and mechanical properties of bone. Collagen exhibits hemostatic properties while also promote bone healing and regeneration. On the other hand, the osteoconductive surface of the calcium phosphate crystals bind non-specifically to negatively charged groups of collagen protein and also prevent aggregation of small hydroxyapatite granules. The collagen-hydroxyapatite has been shown to act as bone void filler in filling buccal gap, ridge preservation, and spinal fusion leading to bone formation in several preclinical and clinical studies [27,28,29,30]. Several Collagen-Hydroxyapatite composites are commercially available. This includes Bio-Oss Collagen, OCS-B Collagen, NuOss Collagen, Ossix bone, Collapat II, Osteon II, Z-core form Xenograft, Osteogen block and others. The most studied composite is Bio-Oss Collagen that consists of 90% bovine hydroxyapatite mixed with 10% porcine collagen indicated for dental bone regeneration. Bio-Oss Collagen implanted in extraction socket showed larger bone socket volume compare to control (untreated) group. In addition, Bio-Oss Collagen showed significant higher bone density in the middle and apical areas of the alveolar bone [25]. A clinical study in humans assessed the vertical and horizontal alterations of buccal alveolar bone after the immediate insertion of an implant together with Bio-Oss^®^ Collagen. It was found that the composite reduced the vertical and horizontal gap by 99.3% and 99.1% respectively at the end of 12 months [30]. When compared to collagen only, the collagen-hydroxyapatite composite formed 339% more new bone in defects grafted with Bio-Oss Collagen in rabbits at the end of Day 14 [31]. Another study in a canine model implanted with the composite displayed less wound shrinkage than the non-augmented defect [32].

Collagen-Hydroxyapatite scaffolds are formed into various shapes that conform to the patient’s defect site. In addition, the scaffold provides excellent structural and mechanical support that is absent in particulate bone graft materials [33]. These composite scaffolds also provide excellent trimming and handling characteristics enhancing their use during oral surgeries.

In this study, we have developed a composite scaffold, InterOss Collagen, combining 10% Type I Collagen of porcine origin with 90% bovine hydroxyapatite granules (weight %). The aim of this study is to compare the physical and chemical characteristics along with local bone implantation effects of InterOss Collagen with a commercially available product, OCS- B Collagen, which has similar material composition. This study shows that the two bone graft substitutes exhibit similar physiochemical properties, though InterOss Collagen shows higher specific surface area and lower compressive strength, properties. We also conducted an in vivo study to evaluate the test device for local tissue responses along with qualitative assessments of percent material resorption and bone formation in the skeletal tissues of New Zealand White rabbits following implantation. We found that the two bone materials are virtually indistinguishable, exhibiting somewhat similar degradation profiles and generated similar inflammatory and healing responses following implantation.

## 2. Materials and Methods

**Preparation of InterOss Collagen**—InterOss Collagen was prepared by mixing bovine hydroxyapatite granules to porcine derived collagen in water in 9:1 ratio (by weight). The resulting slurry was filled in molds, freeze dried (Freezing cycle: −40 °C for 4 h; Primary dry cycle: −10 °C for approx. 33 h and secondary cycle: 10 °C for 4 h) and annealed at 120 °C to arrive at composite scaffolds of cuboids shapes (referred to as blocks) followed by dehydrothermal crosslinking. The resulting scaffolds (hereafter, also referred to as composite blocks or blocks) were used throughout this study.

**Scanning electron microscopy (SEM)**—Thin sections of the composite block were cut using a sterile scalpel and were coated with 3 ∗ 10 nm Pt/Pd coating (coating time 6 s/nm) prior to imaging. SEM images for InterOss Collagen were captured at various magnifications on Tescan GAIA3 SEM/FIB using the built-in software suite. Samples for OCS-B Collagen (blocks) were sectioned and coated with Pt/Pt (5–6 nm) before being imaged using FEI Nova NanoSEM 450.

**Differential Scanning Calorimetry (DSC)**—Heat flow characteristics for InterOss Collagen and OCS-B Collagen samples were measured using differential scanning calorimetry (Shimadzu DSC-60 plus). The DSC curves were recorded in the range 30–300 °C at a heating rate of 10 °C/min for InterOss Collagen and OCS-B Collagen while data for porcine dry collagen was recorded between 30–350 °C.

**Thermogravimetric Analyzer (TGA**)–TGA (Netzsch TG 209 F3 Tarsus) was used to determine the loss in sample mass as a function of temperature under an inert nitrogen atmosphere. TGA curves were taken in the range 25–300 °C at a heating rate of 20 °C/min.

**Compressive strength and Elastic modulus**—To evaluate the strength of the composite grafts, compressive strength and elastic modulus were measured using a Universal Testing Machine (Instron 3369). The dimensions of each specimen were measured using a digital caliper prior to testing. These scaffolds were then placed on compression platens and were subjected to a compressive force at the rate of 1.3 mm/min exerted from the top surface of the scaffolds. Force and displacement data were recorded in order to generate the stress-strain curves. Compressive strength was calculated by dividing the maximum load applied to the original cross-sectional area of the specimen at 50% compressive strain. 

Elastic modulus, a measure of a material’s elasticity or the material’s resistance to non-permanent, or elastic, deformation, was determined using this test. The portion of the curve in the elastic region was used to measure the slope and was determined automatically by the Instron. Bluehill Lite, version 2.29 The software excludes the initial and final portions of the elastic deformation where the stress-strain curve is non-linear. Both compressive strength and elastic modulus were measured simultaneously on the same set of samples (*n* = 5) and the results were displayed in the form of mean ± standard error. 

**Water absorption Capacity Test**—The water absorption test was performed on the two materials- InterOss Collagen and OCS-B Collagen. The samples were weighed using an analytical balance (M_0_) and were then immersed in deionized water (pH = 7.25) for 30 s to achieve complete water absorption. The samples were then removed from the water using tweezers ensuring no drippage and were weighed after (M_1_). The water absorption capacity was calculated using the formula (M_1_ − M_0_)/M_0_.

**Porosity and specific surface area (BET)**—A nitrogen gas adsorption-desorption isotherm was plotted to measure specific surface area using a Micromeritics 3 Flex adsorption analyzer (Micromeritics, Norcross, GA, USA) at −195 °C. Specific surface area was calculated according to Brunauer–Emmett–Teller (BET) method. Pore area, pore diameter, and porosity were measured using MicroActive AutoPore V 9600 analyzer assuming 130° contact angle while pressure applied ranged from 0.2–61,000 psi.

**Fourier Transform Infrared Spectroscopy (FTIR)**—Chemical characterization of InterOss Collagen, OCS-B Collagen and porcine dry collagen was carried out via the Potassium bromide pellet method (sample:Kbr ratio–1:100) and infrared spectra was obtained using FTIR spectroscopy (Nicolet Is5, ThermoFisher Scientific). The spectra of each sample was recorded from 4000 and 400 cm^−1^ with 64 scans at a resolution of 4 cm^−1^.

Each of the tests was tested on at least three samples unless otherwise noted.

**Animal preparation**—Six Adult New Zealand white rabbits (*Oryctolagus cuniculus*) were used for each 2 and 8-week implantation period, while seven rabbits were used for 13-week implantation period. Each implantation period consists of data from an average of at least 10 sites per implant material. Animals were at least 12 weeks old, weighed between 3.05–3.60 kg and were chosen from a large pool of animals to minimize adverse clinical signs. Animals were identified through an ear tattoo and were individually housed in in suspended stainless steel cages at 22 °C and 30–70% humidity throughout the course of the study. Animals were exposed to 12 h light/dark cycle under full spectrum fluorescent lights and were provided bedding, tap water and high fiber diet ad libitum.

**Experimental design and dosage**—InterOss Collagen and OCS-B Collagen were supplied sterile in original packaging for the study. The samples were made wet with sterile saline prior to trimming. Both articles were cut to measure approximately 2.0 mm in width, <1.0 mm in thickness, and 6.0 mm in length before implantation in animals. 

**Creation of implantation site**—Hind leg of each animal was clipped free of fur and loose hair was removed by means of a gentle vacuum. Each animal was appropriately anesthetized before implantation. The lateral cortex of each femur was exposed and then two holes (2.0 mm in diameter) were drilled through the lateral cortex. The femurs receiving the bone graft substitutes had holes drilled at approximately 2.0 mm near the proximal or distal epicondyles. The holes in the left femur of each animal received InterOss Collagen while the holes in the right femur received OCS-B Collagen. A total of at least 10 test article sites and 10 control article sites were implanted on Day 0 for evaluation at each of the three time points—2 weeks, 8 weeks and 13 weeks.

**Post-operative care**—Following implantation surgery, the animals were allowed to recover prior to returning to their cages. Animals were observed daily for the entire period to ensure proper healing of the implant sites and for clinical signs of toxicity. At the end of the observation period, the animals were weighed and sacrificed by an injectable barbiturate.

**Gross observations**—The femurs, containing the test and control articles, were excised *in toto* from the sacrificed rabbits. The surfaces of the excised implant sites were preserved in 10% neutral buffered formalin and were examined macroscopically.

**Histopathology**—Following fixation in formalin, each of the implant sites were excised from the larger mass of tissue. The implant site, containing the implanted material, was examined macroscopically. The bone tissues were decalcified for sectioning and processed for histopathology. Following decalcification, each of the implant sites were excised from the larger mass of tissue leaving at least a 4 mm envelope of surrounding bone tissue. The implant material was left in-situ, and the site was processed. Histological slides of an enface cross−section of the implant site were prepared using routine hematoxylin and eosin stains. The slides were evaluated and graded by light microscopic examination.

**Pathological Assessment of the Effects of the Implant**—The following inflammatory and healing responses were assessed by microscopic observation and the responses graded according to Table 1 and Table 2 for each implant site.

Qualitative assessments for the amount of material remaining and the amount of bone growth in the vicinity of the implant was evaluated based on the scoring scale shown in Table 3.

Authors had no role in selection of animals, sample preparation, imaging, data collection and reporting of the results. Only one pathologist was employed to score all the slides for both OCS-B Collagen and InterOss Collagen. Authors were fully blinded to the implant material as this study was conducted at a contract research organization (Toxikon Corp., Bedford, MA, USA) under compliance with the current US Food and Drug Administration 21 Code of Federal Regulations, Part 58 Good Laboratory Practices (GLP) for Non-clinical laboratory studies. No unforeseen circumstances that affected the integrity of the study were noted. The scores were reported as an average of at least 10 sites for each bone graft material per implantation period. No other statistical analysis was performed on the obtained scores.

## 3. Results

### 3.1. Surface Morphology Using Scanning Electron Microscopy (SEM)

**Figure 1:**
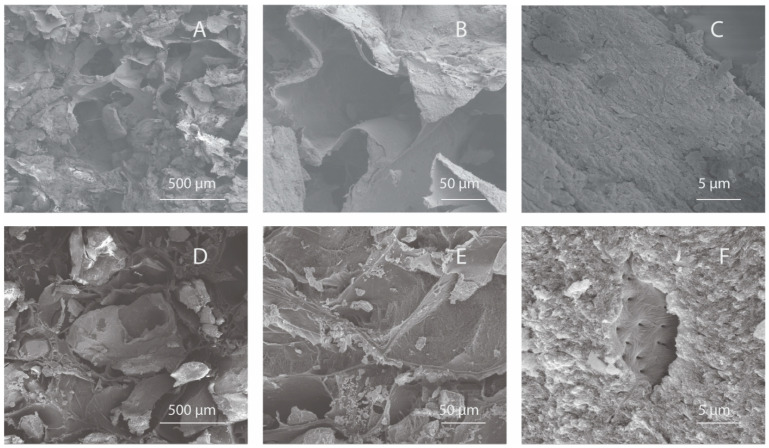
Scanning Electron Microscopy images of InterOss Collagen (**A**–**C**) and OCS-B Collagen (**D**–**F**) taken at various magnifications.

The surface and pore architecture of InterOss Collagen and OCS-B Collagen was studied using scanning electron microscopy (Figure 1). Macroscopically, the shapes of both the scaffolds were similar (cuboid) and displayed irregular distribution of porous structures. SEM analysis of the both bone graft substitutes showed porous microstructure with mineral particles distributed densely throughout the collagen fiber matrix.

### 3.2. Surface Area and Porosity Measurements

InterOss Collagen showed significantly higher specific surface area (77.0 ± 0.2 m^2^/g) than OCS-B Collagen (49.7 ± 1.2 m^2^/g) measured using BET method (Table 4). Mercury Porosimetry revealed that the total pore area, was higher in InterOss Collagen, while porosity and average pore diameter were similar in both scaffolds.

### 3.3. Mechanical Properties

**Figure 2:**
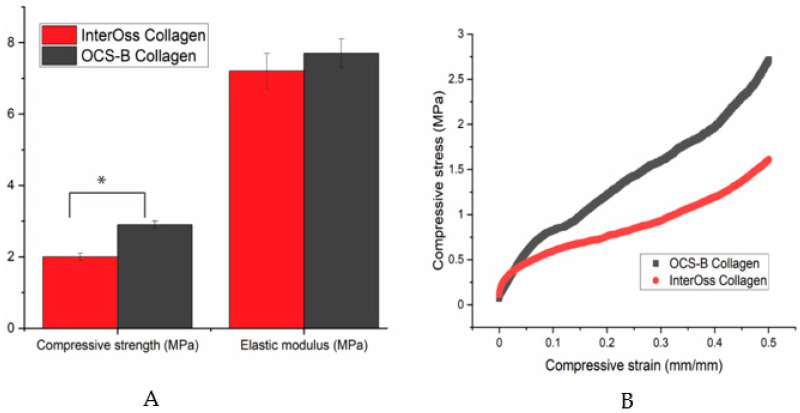
(**A**) A bar graph showing the mechanical properties data and (**B**) A typical stress-strain curve for OCS-B Collagen and InterOss Collagen. Errors bars in SEM (Standard error mean). * indicates statistical significance at 5% (*p* value: 0.03; T-test unequal variance).

A universal compress tester was used to determine the maximum stress a material can sustain under crushing load. Compressive strength was statistically lower in InterOss Collagen (1.81 ± 0.12 MPa) when compared to OCS-B Collagen (2.92 ± 0.14 MPa) at a significance level of 5% (*p* value: 0.03; T-test unequal variance). However, elastic modulus was found to be similar for both materials (Figure 2).

### 3.4. Thermal Properties

Moisture content was measured using a thermogravimetric analyzer (TGA). A TG curve shows percent mass decrement with increasing temperature. The moisture content was found to be 3.1% in InterOss Collagen while it was 2.3% for OCS-B Collagen. For comparison, we also measured the moisture content of the porcine dry collagen, raw material used to manufacture InterOss Collagen, and measured it to be 9.1%. (Table 5). A typical TG curve for InterOss Collagen, OCS-B Collagen, and porcine dry collagen is shown in Supplementary Materials Figure S1.

We measured the heat flow characteristics of InterOss Collagen and OCS-B Collagen using Differential scanning calorimetry. DSC curves showed characteristic broad endothermic peaks: peak 1 at 58 °C (onset temperature 26 °C) followed by a smaller peak between 224–229 °C (onset temperature 214–221 °C) for both bone graft materials (Table 5). These peaks are representative of the two stage sample transformation where peak 1 represents the collagen transition from the triple helix conformation to a random coil structure. The configuration change involves removal of loosely bound water from the molecule and breaking of hydrogen bonds (both intra- and intermolecular) that keeps the helix intact [34]. Peak 2 represents the complex phenomenon of thermal modification involving continued conformational changes to the helical structure along with the release of chemically bound water and small molecule degradation products. The slight difference in the denaturation temperature of InterOss Collagen and OCS-B Collagen in peak 2 could be attributed to inhomogeneous nature of dehydrothermal crosslinking that allows water to evaporate at a varying rate. We also performed thermal analysis on the dry form of porcine collagen and found three endothermic peaks at 65 °C, 204 °C and 304 °C. The two peaks above 200 °C are associated with evaporation of strongly bound water and continued conformational changes to the helical structure of collagen [35]. A typical DSC curve is shown in Supplementary Materials Figure S2.

### 3.5. Water Absorption Capacity

We measured water absorption capacity for the both bone graft substitutes and found InterOss Collagen to exhibit significantly higher water absorption capacity, as compared to OCS-B Collagen. InterOss Collagen exhibited a water swelling ratio of 1.79 compared to 1.16 for OCS-B Collagen, indicating that InterOss Collagen is able to absorb fluid at a faster rate.

### 3.6. Fourier Transform Infrared Spectroscopy (FTIR)

**Figure 3:**
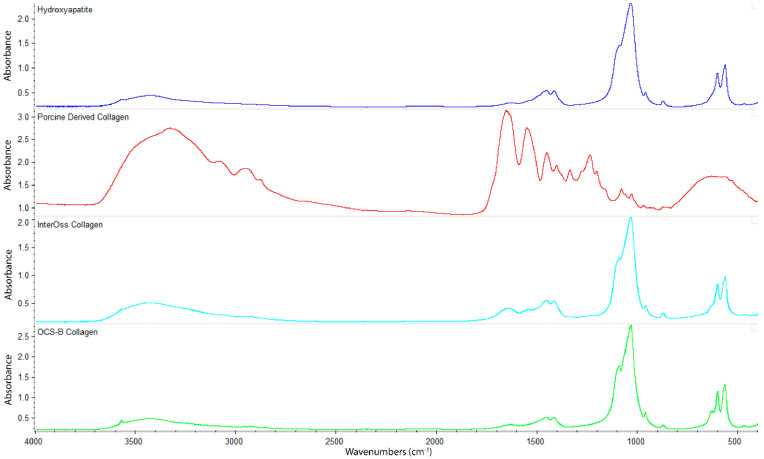
FTIR spectra of Hydroxyapatite, Porcine Derived Collagen, InterOss Collagen, and OCS-B Collagen.

FTIR spectroscopy was employed to determine functional groups of OCS-B Collagen and InterOss Collagen along with latter’s constituent raw materials–Hydroxyapatite and Porcine derived collagen (Figure 3). InterOss Collagen and OCS-B Collagen, composed of collagen and hydroxyapatite, exhibited similar absorption bands with characteristic stretching peaks of CO_3_^2−^ and PO_4_^3^ at 1456, 1415, 1041, and 600–550 cm^−1^. We did not observe distinctive peaks of collagen in InterOss Collagen due to low concentration of collagen in the samples. For comparison, we also obtained spectra on dry collagen, which also showed distinctive amide I and amide II frequencies at 1650 and 1550, respectively, while also exhibiting asymmetric vibrational frequencies at 1450, 1403 and 1239 cm^−1^. Similarly, asymmetric stretching of CO_3_^2−^ groups from hydroxyapatite shows spectral peaks at 1506–1570, 1400–1477 cm^−1^ and 953–989 cm^−1^ [16].

### 3.7. Rabbit Studies

**Figure 4:**
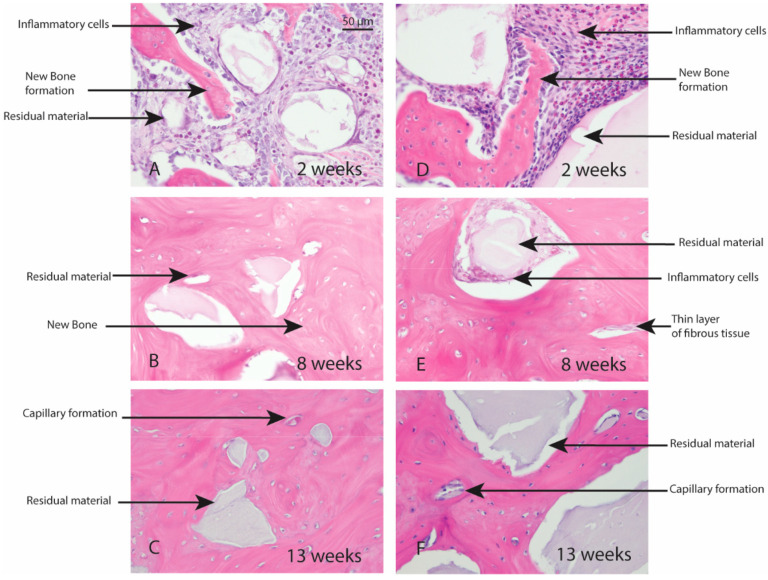
Local effects of implantation in rabbits post placement of InterOss Collagen (**A**–**C**) and OCS-B Collagen (**D**–**F**).

**Figure 5:**
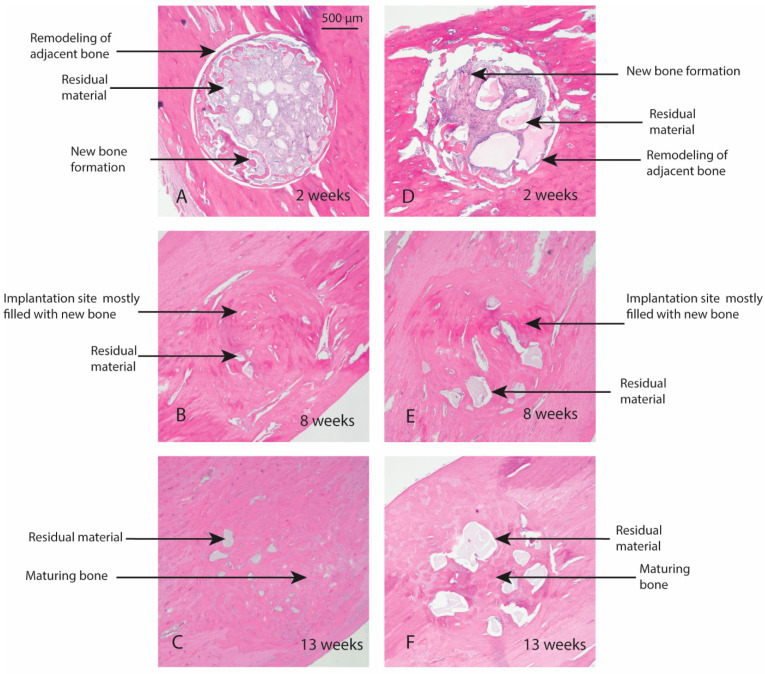
New bone formation in rabbits following implantation of InterOss Collagen (**A**–**C**) and OCS-B Collagen (**D**–**F**).

Macroscopic evaluation of the test article implant sites indicated no significant signs of inflammation, encapsulation, hemorrhage, necrosis, or discoloration at the end of study period. Based on the microscopic inflammatory/healing responses and the amount of residual material remaining/new bone formation, there were no significant differences between InterOss Collagen and OCS-B Collagen (Table 6). InterOss Collagen implantation sites had minimal amounts of residual test material surrounded by maturing bone at the end of 13 weeks (Figure 4). Minimal inflammatory cells and thin bands of fibrosis between test article and surrounding bone were present. Greater than 90% of the cross-sectional area of InterOss Collagen implantation sites were filled with new bone at the end of 13 weeks, with small areas showing marginal bone remodeling (Figure 5). Overall, appearance consistent with complete healing of implantation sites via new bone formation with minimal amounts of residual test material remaining was observed for InterOss Collagen. OCS-B Collagen showed similar new bone formation, inflammatory and healing scores but displayed slightly higher amount of residual material compared to InterOss Collagen at the end of 13 weeks. No evidence that demonstrated local toxic effects in the skeletal tissues of the New Zealand White rabbits was observed throughout the study.

## 4. Discussion

Bone graft substitutes are key bone regenerative materials for dental and orthopedic applications. Their effect on bone regeneration is well-established and several synthetic or biological materials or their composites are commercially available. We have developed a composite scaffold, InterOss Collagen, combining animal-derived collagen and hydroxyapatite granules for bone grafting. We compared the material, and biocompatible properties in this study and determined that overall, InterOss Collagen displays similar physiochemical properties as OCS-B Collagen. The surface area, however, is higher in InterOss Collagen while the compressive strength is lower, which may provide dual advantage of increased surface area for attachment of osteogenic cells while slightly reduced compressive strength improves handling characteristics of the composite. It is known that greater surface area provides robust osteoblast growth and attachment that may lead to higher bone volume [10]. Since resorbable bone grafts such as InterOss Collagen are intended to degrade over 3–6 months, compressive strength is generally considered not as important for performance of functions such as guided bone regeneration compared to inert implants. InterOss Collagen also displayed ~1.5× water absorption capacity than OCS-B Collagen due to enhanced porosity that also aids in ease of trimming during oral surgeries. Based on the FTIR spectra of both OCS-B Collagen and InterOss Collagen, spectral peaks around 1450 cm^−1^ originate from carbonate groups in hydroxyapatite as well as from asymmetric vibrational frequencies of the amino acid groups (–CH_2_). The two peaks may overlap due to non-specific chemical interactions between collagen and hydroxyapatite. We also observed reduced peak intensities due to the presence of collagen [36].

The *in vivo* biocompatibility studies also determined that the two bone graft materials do not demonstrate any significant difference in terms of local effects after implantation. The qualitative assessment revealed that the amount of residual material remaining at the end of 13 weeks was slightly higher for OCS-B Collagen, which suggests marginally faster resorption of InterOss Collagen and may provide the benefit of increased space for new bone formation. Further results of performance of InterOss Collagen in a canine mandibular model along with quantitative assessments using microCT, histologic bone morphometric measurements will be the subject of another publication, which is currently under preparation. Overall, this study concludes that the InterOss Collagen is a promising bone filler composite for applications in augmentation and reconstruction of periodontal defects during oral surgeries.

## Figures and Tables

**Table 1:** Inflammatory responses.

Cell Type/Response	Score				
	0	1	2	3	4
Polymorphonuclear cells	0	Rare, 1–5 phf	5–10 phf	Heavy Infiltrate	Packed
Lymphocytes	0	Rare, 1–5 phf	5–10 phf	Heavy Infiltrate	Packed
Plasma cells	0	Rare, 1–5 phf	5–10 phf	Heavy Infiltrate	Packed
Macrophages	0	Rare, 1–5 phf	5–10 phf	Heavy Infiltrate	Packed
Giant cells	0	Rare, 1–5 phf	5–10 phf	Heavy Infiltrate	Packed
Necrosis	0	Minimal	Mild	Moderate	Severe

**Table 2:** Healing responses.

Cell Type/Response		Score			
	0	1	2	3	4
Neovascularization	0	Minimal capillary, proliferation, focal buds	Groups of 4–7 capillaries with supporting fibroblastic structures	Broad band of capillaries with supporting structures	Extensive band of capillaries with fibroblastic structures
fibrosis	0	narrow band	moderately thick band	thick band	extensive band
fatty infiltrate	0	minimal amount of fat associated with fibrosis	several layers of fat and fibrosis	elongated and broad accumulation of fat cells about the implant site	extensive fat completely surrounding the implant

**Table 3:** Scoring scale for the area involved and bone formation.

Material Remaining Score	Scale	Bone Growth/Formation Score
0 = no material remaining	0	No bone regrowth/formation
1 = up to 25% of defect filled with material	1	1–25% new bone regrowth formation
2 = 25–50% of defect filled with material	2	26–50% new bone regrowth formation
3 = 50–75% of defect filled with material	3	51–75% new bone regrowth formation
4 = greater than 75% of defect filled with material	4	76–100% new bone regrowth formation

**Table 4:** Surface area and porosity measurements.

Properties	InterOss Collagen	OCS-B Collagen
BET surface area m^2^/g	77.0 ± 0.2	49.7 ± 1.2
Total pore area m^2^/g	73.4 ± 1.3	60.2 ± 1.7
Average pore diameter (µm)	0.13 ± 0.0	0.13 ± 0.0
Porosity	79.8 ± 0.4	82.8 ± 0.8

**Table 5:** Thermal properties of InterOss Collagen and OCS-B Collagen.

	InterOss Collagen		OCS-B Collagen		Porcine Dry Collagen		
Moisture (wt.%)	3.1 ± 0.1		2.3 ± 0.4		9.1 ± 0.8		
Peak temperature °C	58.26	224.7	58.28	229.2	65.01	204.93	304.82
Onset temperature °C	26.67	214.7	26.71	221.4	19.65	198.1	275.35

**Table 6:** Qualitative histological scores assessed in rabbits implanted with InterOss Collagen and OCS-B Collagen.

	2-Week		8-Week		13-Week	
	InterOss Collagen	OCS-B Collagen	InterOss Collagen	OCS-B Collagen	InterOss Collagen	OCS-B Collagen
Average score– residual material remaining	2.1	2.3	1.7	2.0	1.4	2.3
Average score– New bone formation	3.1	3.2	3.5	3.3	3.8	3.5
Average Inflammatory Score (polymorphs, lymphocytes, plasma cells, macrophages, giant cells, necrosis)	9.8	10.4	1.6	2.9	0.9	1.3
Average healing score (neovascularization, fatty infiltrate, fibrosis)	2.9	2.7	1.5	2.0	1.7	2.3

## Data Availability

Not applicable.

## Supplementary Materials

The following supporting information can be downloaded at: https://www.mdpi.com/article/10.3390/jfb13010028/s1, Figure S1: A typical DSC curve showing heat flow for InterOss Collagen, OCS-B Collagen and Porcine collagen used to make InterOss Collagen; Figure S2: A typical TG curve representing the percent mass change following the exposure to increasing temperature for InterOss Collagen and OCS-B Collagen.
