# Supplementary material for: A Comparative Study of Two Bone Graft Substitutes—InterOss® Collagen and OCS-B Collagen®
Source: J Funct Biomater. 2022 Mar 9;13(1):28. doi: 10.3390/jfb13010028 (PMC8951741; doi:10.3390/jfb13010028)
Supplement: Supplementary file 1 [file jfb-13-00028-s001.zip › jfb-1621670-supplementary.pdf]

SUPPLEMENTARY INFORMATION

SUPPLEMENTARY FIGURES

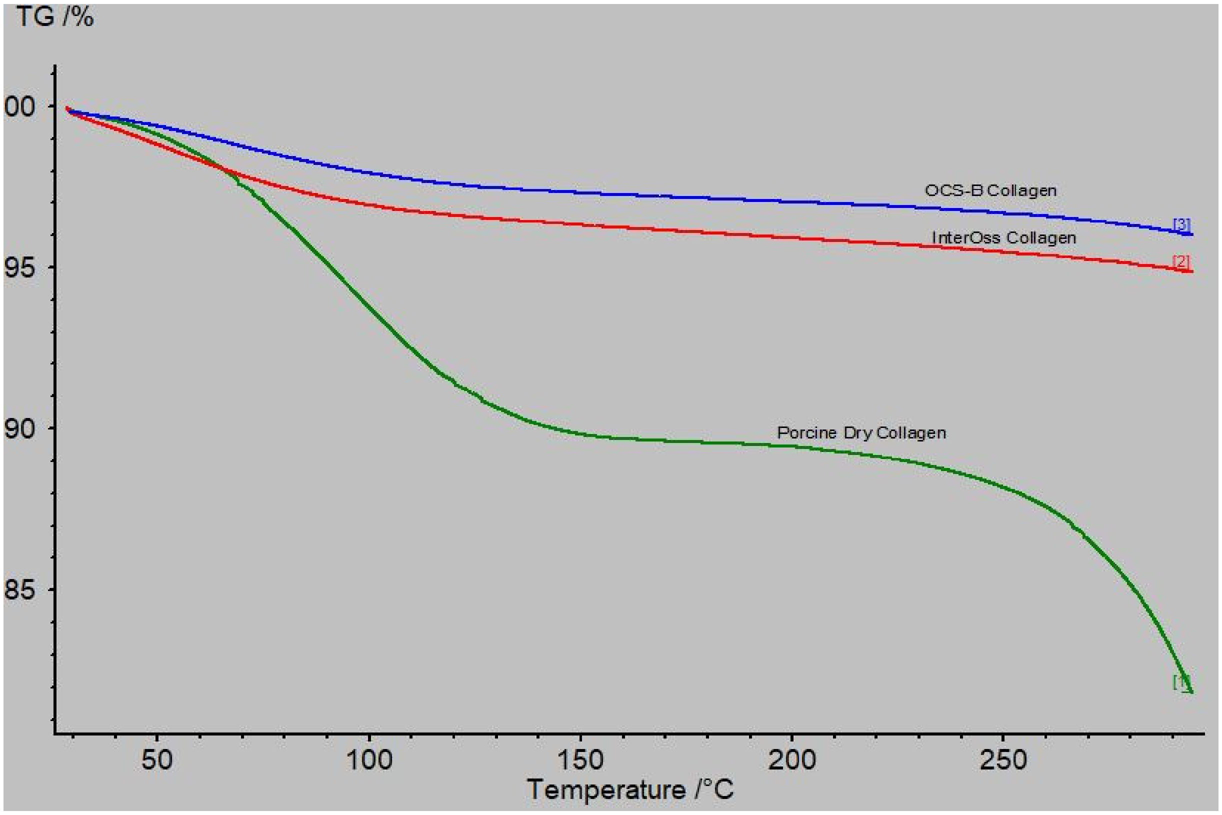

Figure S1 – A typical TG curve representing the percent mass change following the exposure to increasing temperature for InterOss Collagen, OCS-B Collagen and Porcine Dry Collagen.

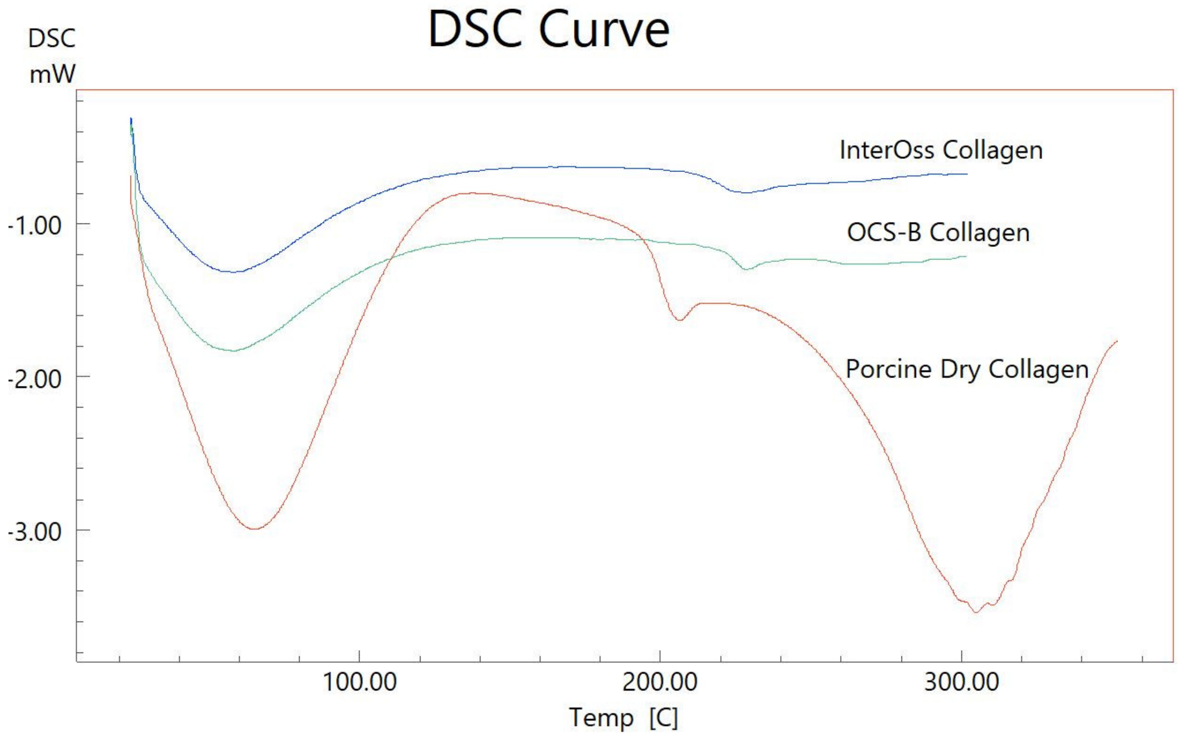

Figure S2 – A typical DSC curve representing heat flow characteristics for InterOss Collagen, OCS-B Collagen and Porcine Dry Collagen.
